# Corrosion Resistance of Calcium Aluminate Cement Concrete Exposed to a Chloride Environment

**DOI:** 10.3390/ma7020887

**Published:** 2014-01-28

**Authors:** Ki Yong Ann, Chang-Geun Cho

**Affiliations:** 1 Department of Civil and Environmental Engineering, Hanyang University, Ansan 426, Korea; E-Mail: kann@hanyang.ac.kr; 2 School of Architecture, Chosun University, Gwangju 501, Korea

**Keywords:** calcium aluminate cement, corrosion, chloride, setting

## Abstract

The present study concerns a development of calcium aluminate cement (CAC) concrete to enhance the durability against an externally chemically aggressive environment, in particular, chloride-induced corrosion. To evaluate the inhibition effect and concrete properties, CAC was partially mixed with ordinary Portland cement (OPC), ranging from 5% to 15%, as a binder. As a result, it was found that an increase in the CAC in binder resulted in a dramatic decrease in the setting time of fresh concrete. However, the compressive strength was lower, ranging about 20 MPa, while OPC indicated about 30–35 MPa at an equivalent age. When it comes to chloride transport, there was only marginal variation in the diffusivity of chloride ions. The corrosion resistance of CAC mixture was significantly enhanced: its chloride threshold level for corrosion initiation exceeded 3.0% by weight of binder, whilst OPC and CAC concrete indicated about 0.5%–1.0%.

## 1. Introduction

Calcium aluminate cement (CAC) was initially developed to protect from external aggressive chemicals by reducing the hydration of Ca(OH)_2_, containing a high portion of Al_2_O_3_ in the oxides, accounting for 50%–60%. The hydration process of CAC concrete is sensitively governed by curing temperature, thereby leading to a rapid development of strength even at an early age, often accounting for 40–50 MPa in a few days. Notwithstanding, its use for structural concrete has been limited due to a reduction of strength in a relatively hot environment, of which the risk has been, however, challenged. A very rapid development of the strength may compensate for the strength reduction at 20–60 days, since the strength gained after the reduction period is usually still higher than for ordinary Portland cement (OPC) concrete [1].

Moreover, the CAC has benefits in raising the durability of concrete against a chemically corrosive environment, as the CA hydration (*i.e.*, C_12_A_7_) is resistive against reacting with external chemicals [2]. In particular, a high level of Al_2_O_3_ in oxides of CAC has led to a possibility of inhibition of steel concrete in concrete, as C_3_A in hydration consisting of Al_2_O_3_ and CaO binds chlorides in the pore solution then to remove from the corrosion process. Likewise, a current study showed that CAC concrete had a slightly higher resistance to the onset of corrosion, when immersed in an NaCl solution [3], presumably due to immobilization of chloride ions even after the acidification of the pore solution at pit nucleation [4]. In a previous study, the corrosion behavior of CAC concrete was more passive than OPC, evaluated by the corrosion rate in terms of polarization resistance, depending on the curing temperature [5].

Alternatively, a mixture of CAC and OPC was suggested to mitigate the expense rise, and simultaneously to sustain the benefit of CAC concrete. The mixture has, however, a limitation in mixing and casting of fresh concrete, due to a potential risk of flash set. Unlike the setting of Portland cement, the CAC mixture with OPC may set in a few minutes after mixing, as being less practical in *in-situ*. The ratio of CAC to OPC for the flash set is often from 3.0: 7.0 to 7.0: 3.0 [6], as being risky of heat generation, leading to thermal cracking and lower strength development. For the durability of the CAC mixture, it was reported that the corrosion resistance was very high in terms of the chloride threshold level for corrosion; the chloride threshold value for the CAC mixture exceeded 2.0% by weight of binder, depending on the air void content at the steel-concrete interface [7,8]. Also, a mixture of CAC with zeolite had a prominent inhibition of steel in a chloride environment, when assessed by visual examination [9]. However, the inhibitive nature of the CAC mixture was not clearly known, for example, chloride binding or inhibitive layer generation on the steel surface, to date.

In the present study, to identify the inhibition of CAC concrete, corrosion test including corrosion potential, chloride binding capacity and chloride transport were measured together with concrete properties (strength and setting time). In particular, a CAC mixture with OPC was intensively tested. The ratio of CAC to OPC is ranged from 5% to 15% to total binder. For the corrosion potential measurement, a mortar specimen with centrally located steel bar was cast, while the chloride binding was measured using paste. Chloride transport was evaluated by the diffusivity of chloride ions in concrete, when exposed to a salt solution. In the curing and storing process, the temperature was kept at 20 °C to avoid the influence of temperature.

## 2. Experiments

To investigate the inhibitive effect of CAC concrete, corrosion resistance was quantitatively measured by the electrochemical techniques, together with the rate of chloride transport and chloride binding capacity. Simultaneously, mixtures of CAC and OPC were fabricated to assess their inhibition effect, within the range of 5%, 10% and 15% of CAC to the total binder content. The oxide composition of CAC and OPC is given in Table 1.

Mix proportion for paste, mortar and concrete is given in Table 2. Cement paste was used for testing of chloride binding, mortar for the corrosion resistance and concrete for fundamental properties and chloride transport, respectively. A free water/binder ratio was always kept at 0.4.

### 2.1. Fundamental Properties

The compressive strength of concrete was measured with cylindrical specimens (Ø100 × 200 mm), at 7, 28, 56 and 91 days. Concrete specimens were demolded 24 h after casting and then cured in a 95% humid chamber at 20 ± 2 °C. The setting time was determined by the penetration resistance of fresh concrete. A mortar specimen was obtained by sieving fresh concrete with the 5.0 mm sieve to remove gravel immediately after the completion of concrete mixing. Then the mortar was placed in a cylindrical mold (Ø300 × 400 mm), followed by a subsequent vibration to remove air bubbles in concrete. The penetration resistance was measured at a given time interval. The initial set was defined as the time for the penetration resistance to reach 3.43 MPa, and the final set to 27.46 MPa.

### 2.2. Chloride Binding

In the testing of chloride binding, cement paste was cast, as aggregates do not affect the chemistry between cement paste and aggregate. Six levels of chlorides were admixed in mixing water as NaCl: 0%, 0.5%, 1.0%, 1.5%, 2.0% and 3.0% by weight of binder. After casting the paste, the specimen was rotated at 6.0 rpm for 24 h to avid segregation of chloride ions, and then cured for 56 days by wrapping in a polythene film at 20 2 °C to avoid a leaching-out of ions in the cement matrix. Prior to measuring the concentration of chloride ions in the cement paste, the specimen was dried in an oven at 104 °C for 24 h and then crushed/ground to obtain dust sample, which was then sieved into the 300 μm sieve in fineness. The dust sample was stirred for 5.0 min in 50 °C distilled water to extract the water-soluble chloride [2]. After a further 30 min standing of the sample, the concentration of chloride ions in the cement paste was measured by the potentiometric titration against silver nitrate. Then, the binding capacity was identified by the ratio of non-water soluble chloride to total chloride concentration.

### 2.3. Chloride Transport

The rate of chloride transport was determined by the chloride diffusivity in concrete exposed to saturated salt solution. After 28 days curing of concrete in a humid chamber at 20 ± 2 °C, the specimen was cut to 50 mm in the thickness, followed by coating the specimen with epoxy resin, except one surface for chlorides to penetrate the concrete in one direction. Then, the specimen was immersed in the 4 M NaCl solution for 150–180 days before chloride profiling. The chloride profiles were obtained from samples collected by grinding the concrete surface in 2.0 mm depth increments. The acid soluble chloride concentration at all depths was measured by potentiometric titration against silver nitrite. The concentration of the surface chloride (*C_S_*) was determined by the best fitting and the apparent chloride diffusion coefficient (*D*) was fitted to the error function solution to the Fick’s second law, for non-steady state diffusion in a semi-infinite medium, given by Equation (1).

$$C(x,t)=C_{S}(1-erf\frac{x}{2\sqrt{Dt}})$$ (1)

where, the *C* is for the chloride concentration at the depth of *x*; at the time of *t* and *D* for the diffusivity of chloride ions. The time of exposure to a salt solution was taken into account in calculating the diffusivity of chloride ions.

### 2.4. Corrosion Behavior

The corrosion of steel in concrete was measured by half-cell potential using the calomel electrode then to quantify the corrosiveness of steel embedment. In fact, the threshold voltage for the onset of corrosion accounts for −275 mV *vs*. SCE. Six levels of chlorides were admixed in mortar specimens with a centrally located 10.0 mm diameter mild steel bar: the chloride concentration added in the specimens ranged 0%, 0.5%, 1.0%, 1.5%, 2.0% and 3.0% by weight of cement. The two ends of the steel bars were masked using rich cement paste, followed by further covering with heat shrink insulation to avoid corrosion under the masking. The specimens were cured by wrapping in a polythene film for 28 days. To accelerate the corrosion process, the specimens were subjected to a 4 days wet and 3 days of dry conditions. During the wet duration, the relative humidity was kept 95% at 50 °C, while for the dry condition, it was 50% RH at 20 °C. The corrosion potential was measured immediately after the completion of every wet cycle to minimize the electrical resistance of mortar.

## 3. Results

### 3.1. Development of Strength

**Figure 1.**
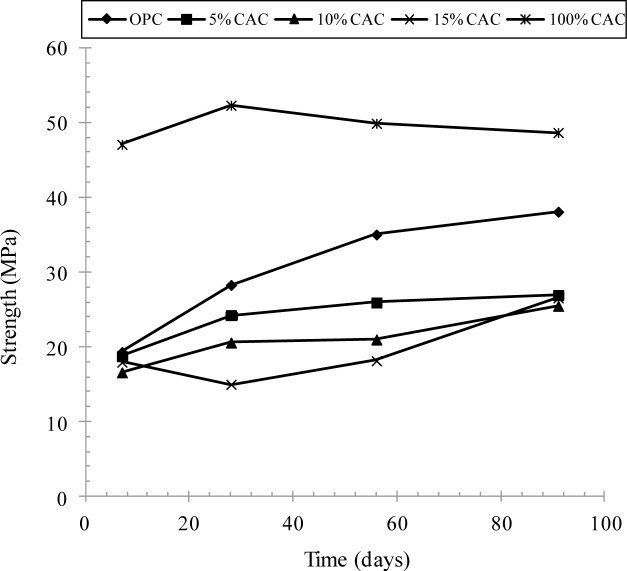
Development of compressive strength for mixtures of CAC and OPC.

A development of the compressive strength for concrete containing the CAC was measured at 7, 28, 56 and 91 days, as given in Figure 1. The strength for CAC concrete was the highest at all ages. It is notable that that the compressive strength was rapidly increased for early ages, reaching beyond 52 MPa at 28 days, and then reduced with time up to about 48 MPa, while OPC concrete indicated a gradual increase, about 30–35 MPa at the equivalent ages. A rapid development of the strength for CAC concrete may be attributed to the CA-type hydrations (e.g., C_12_A_7_, CAH_10_), which could accelerate the hydration process at an early age. A reduction of the compressive strength seems presumably due to the conversion process in hydration from the hexagonal CAH_10_ phase to the cubic C_3_AH_6_ phase, accompanying an increase in the porosity in the cement matrix [10]. The concrete mixed with OPC and CAC produced a lower strength at all ages depending on the mix ratio of CAC: an increase in the CAC in binder resulted in, in fact, a lower strength at a given age. For example, the strength for 5% CAC concrete was 25 MPa at 28 days, and only 15 MPa of the strength was achieved for 15% CAC concrete. The lower strength for CAC mixture is presumably associated with a very rapid setting of fresh concrete [5].

**Figure 2.**
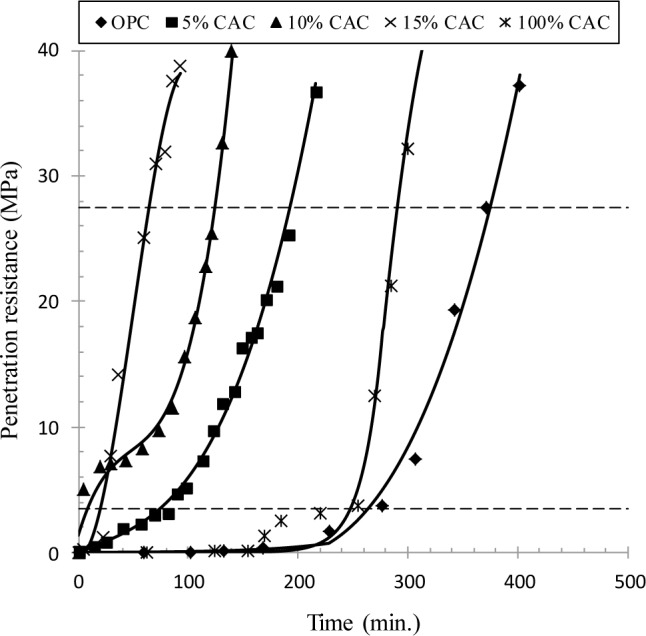
Penetration resistance of fresh concrete with time to determine the setting time.

The penetration resistance of fresh concrete with time is given in Figure 2, to determine the setting time of concrete. An increase in the penetration resistance was best fitted to define the initial and final setting times. For OPC and CAC concrete, the final set was achieved at 375 and 304 min respectively. However, a mixture of OPC and CAC dramatically reduced the setting time, depending on the CAC content in binder. The final set for 5%, 10% and 15% CAC was achieved at 193, 125 and 65 min respectively. A rapid setting for the CAC mixture, in fact, may impose adverse effect to concrete properties, such as thermal cracking and thus lower strength as seen in Figure 1. Thus, a further treatment, for example chemical retarder, seems required to meet the concrete quality, and to secure the time of concrete casting and placing in *in-situ.* In the present study, a study for chemical retarders was not dealt with.

### 3.2. Removal of Chlorides

The presence of chlorides in the cement matrix was evaluated by chloride binding. After 56 days of curing, to fully hydrate cement paste, the concentration of water soluble chloride in the cement paste was determined at a given total chloride concentration, then to derive the binding capacity (ratio of non-water soluble to total chlorides). It is evident that a mixture of CAC and OPC had an even higher binding capacity, compared to OPC paste. Except for CAC paste, the binding capacity was reduced with an increase in the total chloride concentration in the cement matrix and then converged to about 0.68–0.72 with chloride concentration. The binding capacity for CAC paste was lower, accounting for about 0.60 at 1.5%–3.0% of chlorides. Additionally, it was seen that CAC less bound chlorides at the lower chloride concentration, ranging 0.5%–1.0%, at which other binders showed the highest binding capacity.

### 3.3. Mobility of Chloride Ions

**Figure 3.**
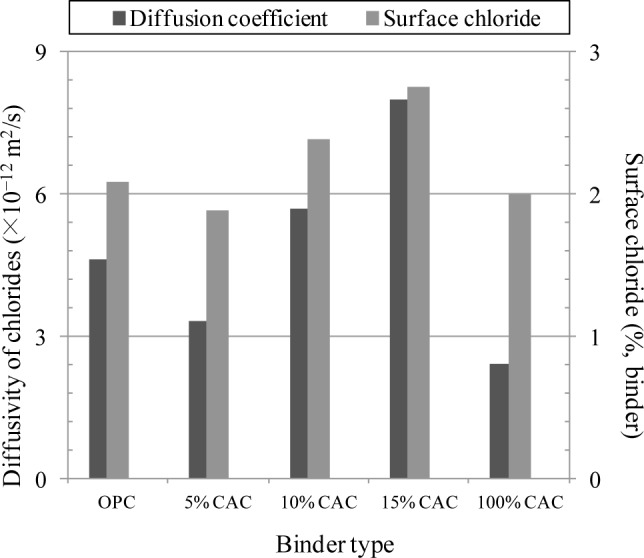
Rate of chloride transport in terms of ionic diffusion with surface chloride.

The diffusivity of chloride ions was calculated for concrete specimens immersed in a 4.0 M NaCl solution for 150–180 days. The diffusivity and surface chloride concentration were calculated using the chloride profiles with 2.0 mm, as given in Figure 3. It is evident that the variation in the diffusion coefficient was marginal; but the diffusivity for CAC concrete was the lowest range of 2.41 × 10^−12^ m^2^/s, whilst the OPC produced the diffusivity accounting for 63 × 10^−12^ m^2^/s. For the mixture of CAC and OPC, the diffusivity was in the similar range of OPC and CAC concrete, ranging from 3.32 × 10^−12^ to 8.00 × 10^−12^ m^2^/s. For the surface chloride concentration, OPC and CAC indicated 2.09% and 2.00% by weight of binder, while the CAC mixture produced slightly higher surface chloride, ranging from 1.88% to 2.75%.

### 3.4. Corrosion Resistance

**Figure 4.**
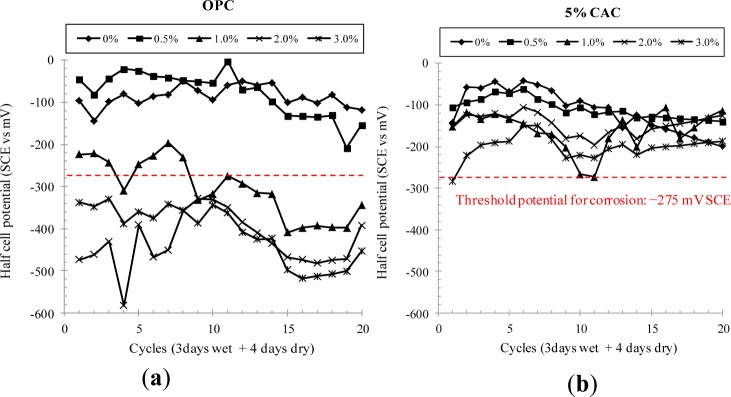

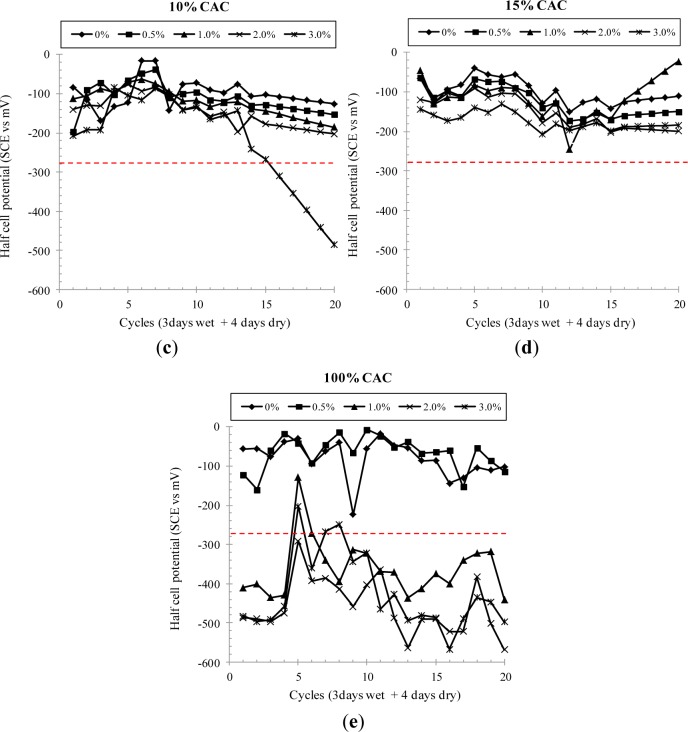
Corrosion potential of steel in CAC and OPC mortar subjected to chloride environments. (**a**) OPC; (**b**) 5% CAC; (**c**) 10% CAC; (**d)** 15% CAC and (**e**) 100% CAC.

The corrosion behavior of steel in CAC and OPC mortar containing the variation in chloride concentration was measured by the half-cell potential method. To accelerate the corrosion process, the specimens were subjected to a repetitive wet and dry cyclic condition for 20 cycles, and the corrosion rate and potential were measured every cycle after the completion of wet duration. As seen in Figure 4, the corrosion potential was strongly influenced by the binder type, chloride concentration in cast and duration of cycles. For OPC specimens, the corrosion potential was lower than the corrosion threshold voltage (*i.e.*, −275 mV *vs*. SCE), when chlorides in cast exceeded 1.0% by weight of binder. This was again observed for CAC mortar, implying that these two binders may have about 0.5%–1.0% of the critical chloride concentration for the onset of corrosion. For the mixture of CAC and OPC, however, the corrosion potential did not indicate any sign of corrosion initiation at all levels of chlorides for all duration, except at 3.0% of chlorides for 10% CAC mortar.

## 4. Discussion

### 4.1. Prevention of Steel Corrosion

**Figure 5.**
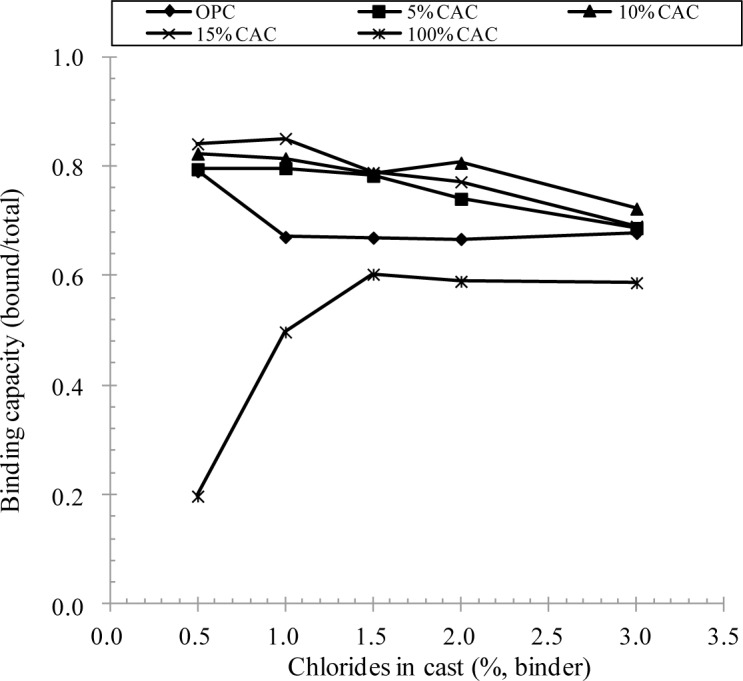
Binding capacity of chlorides in CAC and OPC paste.

In the present study, a mixture of OPC and CAC had an even higher resistance to corrosion of steel, whilst CAC alone had a marginal change in the corrosion behavior in spite of a rapid development of concrete strength. The inhibition effect of CAC mixture may arise from an increase in the chloride binding capacity; chloride binding mitigates the mobility of chloride ions in the pore solution, and thus the binding capacity has been taken as an inhibition parameter. Once chlorides are bound in the cement matrix in terms of immobilization, chloride ions bound in hydration products are in fact removed from the corrosion process. As seen in Figures 3 and 5, the chloride binding capacity for the CAC mixture was even higher than for OPC and CAC solely mixed specimens, thereby leading to a higher resistance to corrosion. Although the chloride binding for CAC was always, however, lower than for OPC, the corrosion resistance was higher. It may suggest that the onset of corrosion may be affected by more influencing factors, such as the acid neutralization capacity (*i.e.*, buffering to a pH fall).

In Portland cement concrete, bound chlorides may be mostly released into free by a fall in the pH of the pore solution in the vicinity of steel [11] and turn mobile to participate in the corrosion process. As the buffering of hydration products to acidification of the pore solution is dependent on binder type, a high buffering capacity is always accompanied by the low risk of pitting corrosion [12]. An experimental study showed that the release of bound chlorides in CAC paste was marginal when the specimen was exposed to a carbonation environment [13]. Moreover, a mixture of CAC and OPC forms a different type of crystallized hydration products, which can strongly trap chloride ions in the matrix.

In a previous study on the semi-quantitative analysis of XRD, no Friedel’s salt was observed for OPC paste, whereas CAC paste still contained bound chlorides at a large portion even subjected to acidification [4]. Substantially it can be said that chlorides bound in the CAC matrix may be less released into free against a fall in the pH. In the majority of guidelines, it is advised that the maximum concentration of chloride ions at the depth of the steel must not exceed 0.2% or 0.4% by weight of binder [14,15], whilst the chloride threshold level for the onset of corrosion ranges from 0.4% to 1.2% by weight of binder [16]. In this study, it was found that the chloride threshold level is in the range of 0.5%–1.0% for OPC and CAC concrete, assessed by the half-cell potential method. In fact, CAC concrete may not provide any benefit in enhancing the chloride threshold level, apart from other advantages (*i.e.*, strength development).

However, a mixture of CAC and OPC showed a prominent higher chloride threshold level for all specimens, exceeding 3.0% by weight of binder. It may suggest that CAC mixture is definitely free from the risk of chloride-induced corrosion, considering that the surface chloride concentration is usually ranged from 2.0% to 2.5% for concretes directly exposed to seawater. When it comes to the Fick’s in calculating the time to corrosion, 1.5%–2.0% of the chloride threshold level, in fact, imposes several hundreds years of the corrosion-free life of concrete structures as long as the rate of chloride transport is assumed to OPC concrete (*i.e.*, < 10^−12^ m^2^/s).

### 4.2. Benefits of CAC Concrete

CAC was initially developed to avoid gypsum-bearing water on Portland cement, consisting of monocalcium aluminate (*i.e.*, CA). The hydration of CA drives the highest rate of strength development: at the low temperature (< 15 °C), the hexagonal CAH_10_ phase is formed by hydration, while the cubic phase of C_3_AH_6_ is produced in the hydration process at the high (>60 °C) temperature. The hexagonal CAH_10_ phase, a dense matrix against porosity, is transformed to the cubic C_3_AH_6_ phase and alumina gel then to form the stable crystal structure. In this conversion process, the volume of the phase is decreased, which in turn forms further porosity in the matrix, and thus the strength of CAC concrete may significantly decrease. However, it does not seem that the conversion process may be problematic in strengthening CAC concrete. Arya [1] currently showed that the strength of CAC concrete was rapidly increased at an early age (within 28 days), but decreased for a while. Then, the strength had neither further increase nor decrease at room temperature. This phenomenon was ensured in the present study that the strength of CAC concrete cured at 20 °C was significantly increased at 7 and 28 days, decreased at 56 days, and no further change in the strength was eventually observed. However it is notable that a reduction of strength for CAC mixture might be problematic: its strength ranged around 20 MPa, while OPC showed 30–35 MPa.

Notwithstanding, a CAC mixture is still beneficial for structural concrete, it is very resistive against chemical attack. As already mentioned, a higher corrosion resistance is attributed to binding of chloride ions, which could increase the chloride threshold level presumably beyond 3.0% by weight of binder. Moreover, CA-hydrations in the CAC matrix may accelerate adorption of Ca(OH)_2_, of which precipitation is removed, as being more resistive to aggressive chemical environments. For example, the absence of Ca(OH)_2_ hydrations may minimize the risk of sulfate attack, which must accompany Ca(OH)_2_ in the cement matrix to react with sulfate ions to produce expansive ettringite [17,18]. The benefit of CAC mixture may be associated with a rapid setting time of fresh concrete, of which value can be reduced to 3.0 h or below then to develop a very early strength of concrete. Otherwise, a chemical retarder must be mixed to secure time of placing fresh concrete, if necessary.

## 5. Conclusions

In this study, CAC concrete was mainly dealt with, in particular, its strength development, setting time and chloride-induced corrosion of steel. Simultaneously, a mixture of CAC with OPC, as a binder, was tested. The conclusion derived from the present experimental study is as follows:

(1) A mixture of CAC and OPC ranked a very rapid setting of fresh concrete depending on the CAC portion. However, its strength development was always lower than for OPC and CAC alone, due to an extremely rapid setting of concrete, of which cause for the lower strength was not clearly identified in the present study. It is notable that CAC solely mixed concrete ranked the highest strength at all ages in spite of a reduction of the strength after 28 days.
(2) The rate of chloride transport in terms of chloride diffusivity was not affected by CAC binder: in fact, the diffusivity of chloride ions and surface chloride concentration were not much different with binder type. The diffusivity of chlorides in concrete ranged from 2.41 × 10^−12^ to 8.00 × 10^−12^ m^2^/s, while the surface chloride was in the range of 1.88%–2.75% by weight of binder.
(3) Chloride binding capacity was strongly dependent on binder type and the amount of CAC in binder. An increase in the CAC content resulted in an increase in the chloride binding capacity, but CAC solely mixed paste produced the lowest chloride binding. Increased binding capacity may enhance the corrosion resistance of CAC mixture
(4) A mixture of CAC has a very high resistance to chloride-induced corrosion of steel, compared to OPC and CAC solely mixed specimens. For the CAC mixture, no corrosion symptom was observed by monitoring of the half-cell potential, whilst OPC and CAC indicated the onset of corrosion at 0.5–1.0 of chlorides in cast. This may arise from increased chloride binding capacity and presumably buffering to a pH fall of the pore solution. The chloride threshold level for the CAC mixture exceeded 3.0% by weight of binder.

## Figures and Tables

**Table 1.** Oxide composition of calcium aluminate cement (CAC) and ordinary Portland cement (OPC) (%).

Oxide	CaO	SiO_2_	Al_2_O_3_	Fe_2_O_3_	MgO	Na_2_O	K_2_O	SO_3_
CAC	28.5	0.2	71.0	0.1	0.4	0.2	–	–
OPC	63.8	22.1	5.0	3.0	1.6	0.35	0.64	2.0

**Table 2.** Mix proportion for paste, mortar and concrete.

Type	Binder	Water	Sand	Gravel	Experiments
paste	1.00	0.40	–	–	chloride binding
mortar	1.00	0.40	2.45	–	corrosion potential
concrete	1.00	0.40	2.45	3.17	concrete strength setting time chloride transport
